# Supplementary material for: Fluorine NMR study of proline-rich sequences using fluoroprolines
Source: Magn Reson (Gott). 2021 Nov 9;2(2):795–813. doi: 10.5194/mr-2-795-2021 (PMC10539733; doi:10.5194/mr-2-795-2021)
Supplement: The supplement related to this article is available online at: https://doi.org/10.5194/mr-2-795-2021-supplement. [file mr-2-795-supplement.zip › mr-2-795-2021-supplement-title-page.pdf]

Supplement of Magn. Reson., 2, 795–813, 2021
https://doi.org/10.5194/mr-2-795-2021-supplement
© Author(s) 2021. CC BY 4.0 License.

Supplement of

Fluorine NMR study of proline-rich sequences using fluoroprolines

Davy Sinnaeve et al.

Correspondence to: Bruno Kieffer (kieffer@igbmc.fr) and Davy Sinnaeve (davy.sinnaeve@univ-lille.fr)

- mr-2-795-2021-supplement-title-page.pdf
- MR-2021-54_SuppMat
  - MR-2021-54-nb0.ipynb
  - MR-2021-54-nb1.ipynb
  - MR_fPRO_Supp_Mat_210909.pdf

The copyright of individual parts of the supplement might differ from the article licence.
