# Supplementary material for: Fluorine NMR study of proline-rich sequences using fluoroprolines
Source: Magn Reson (Gott). 2021 Nov 9;2(2):795–813. doi: 10.5194/mr-2-795-2021 (PMC10539733; doi:10.5194/mr-2-795-2021)
Supplement: The supplement related to this article is available online at: https://doi.org/10.5194/mr-2-795-2021-supplement. [file mr-2-795-supplement.zip › MR-2021-54_SuppMat/MR_fPRO_Supp_Mat_210909.pdf]

Fluorine NMR study of proline-rich sequences using fluoroprolines

Davy Sinnaeve[1,2], Abir Ben Bouzayene[3], Emile Ottoy[4], Gert-Jan Hofman[4,5], Eva Erdmann[3], Bruno Linclau[5], Ilya Kuprov[5], José C. Martins[4], Vladimir Torbeev[6] and Bruno Kieffer[3]

[1]Univ. Lille, Inserm, Institut Pasteur de Lille, CHU Lille, U1167 – Labex DISTALZ – RID-AGE – Risk Factors and Molecular Determinants of Aging-Related Diseases, F-59000 Lille, France

[2]CNRS, ERL9002 - Integrative Structural Biology, F-59000 Lille, France

[3]Departement of Integrative Structural Biology, IGBMC, Université de Strasbourg, INSERM U1258, CNRS UMR7104, 1, rue Laurent Fries, F-67404 Illkirch, France

[4]Department of Organic and Macromolecular Chemistry, Ghent University, Campus Sterre, S4, Krijgslaan 281, B-9000 Gent, Belgium

[5]School of Chemistry, University of Southampton, Southampton SO17 1BJ, United Kingdom

[6]Institut de Science et d'Ingénierie Supramoléculaires (ISIS), International Center for Frontier Research in Chemistry (icFRC), University of Strasbourg, CNRS UMR 7006, F-67000 Strasbourg, France

Supplementary Material

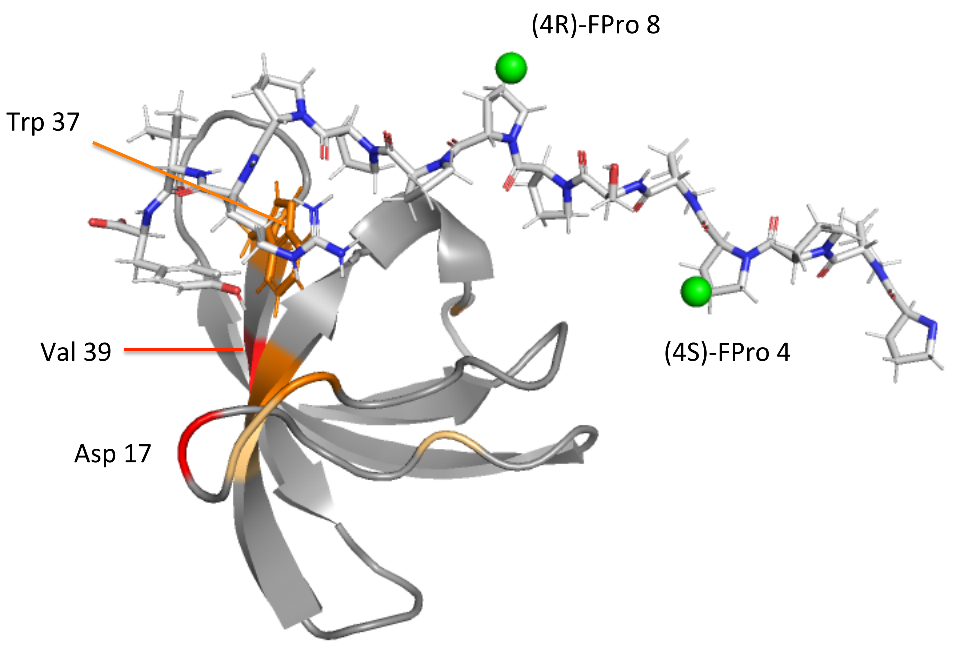

Supplementary Figure 1: 3D homology model of a complex between the MpSR model peptide and the SH3.3 domain of Vinexin β. The structure of the SH3.3 domain was built using the structure of the homologous SH3.1 domain from the same protein (PDBID 2CT3) using MODELLER. The position of the ligand peptide was infered from the structure 1PRL with sequence AFAPPLPRR. The polyproline segments were built assuming a PPII conformation with dihedral angles of -75° and 145° for phi and psi, respectively. The figure was made using Pymol. The residues displaying the largest chemical shift perturbations are colored from light orange to red according the importance of the perturbation.

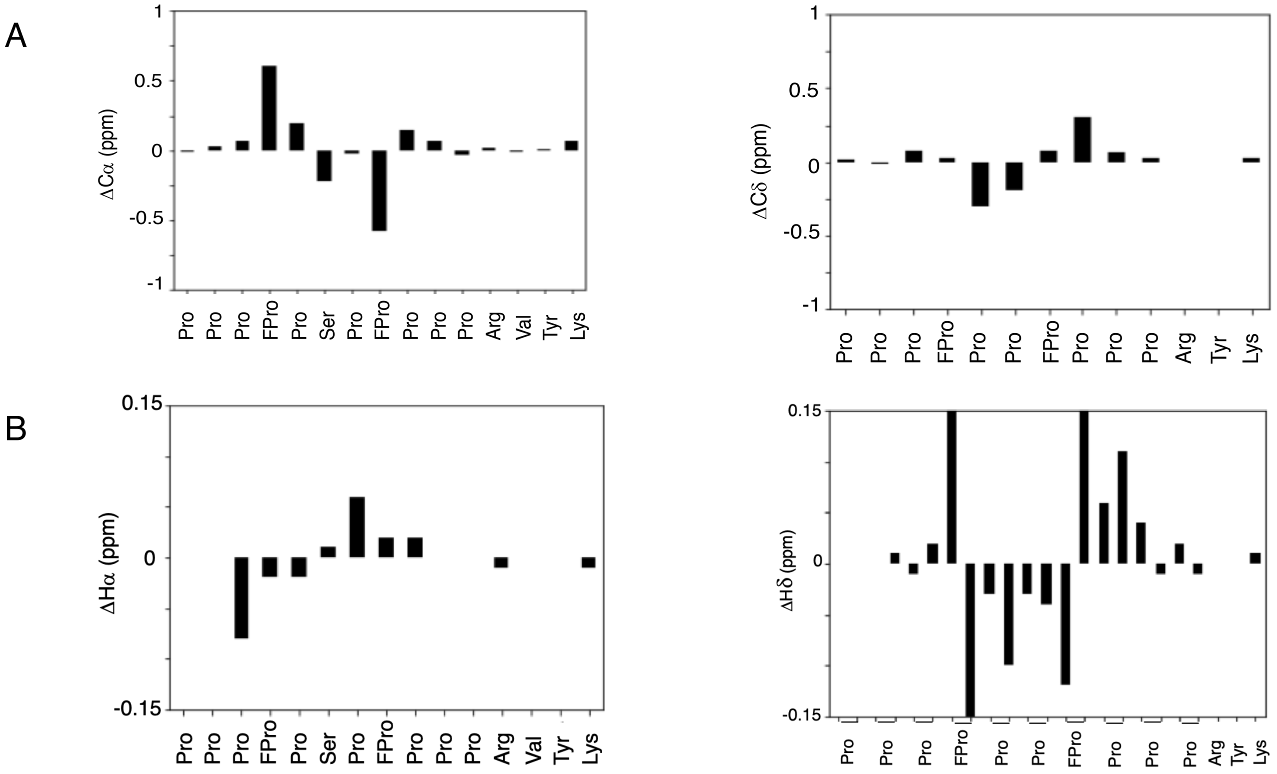

Supplementary Figure 2: Comparison of chemical shifts measured for MpRS and MpSR peptides in water at 298K at 600 MHz. **A:** ^{13}C Chemical shift difference between MpRS and MpSR at positions alpha (left) and delta (right). **B:** ^{1}H Chemical shift difference between MpRS and MpSR at two positions alpha (left) and delta (right).

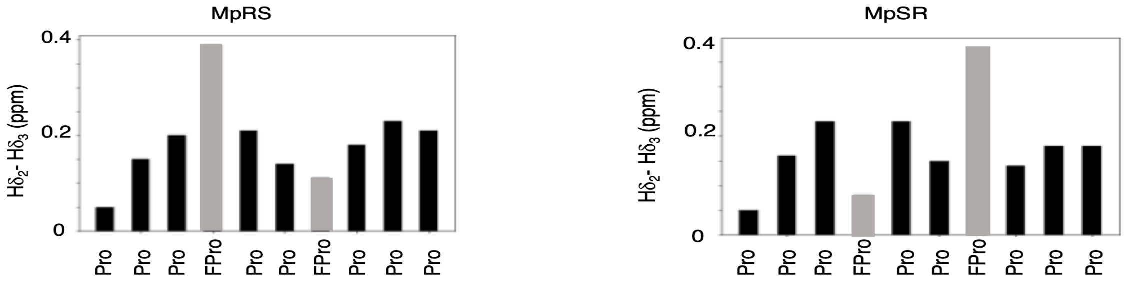

Supplementary Figure 3: Comparison of delta geminal protons chemical shifts differences measured for the MpRS and MpSR peptides in water at 298K at 600 MHz. Fluoroprolines at positions 4 and 8 are shown by gray bars.

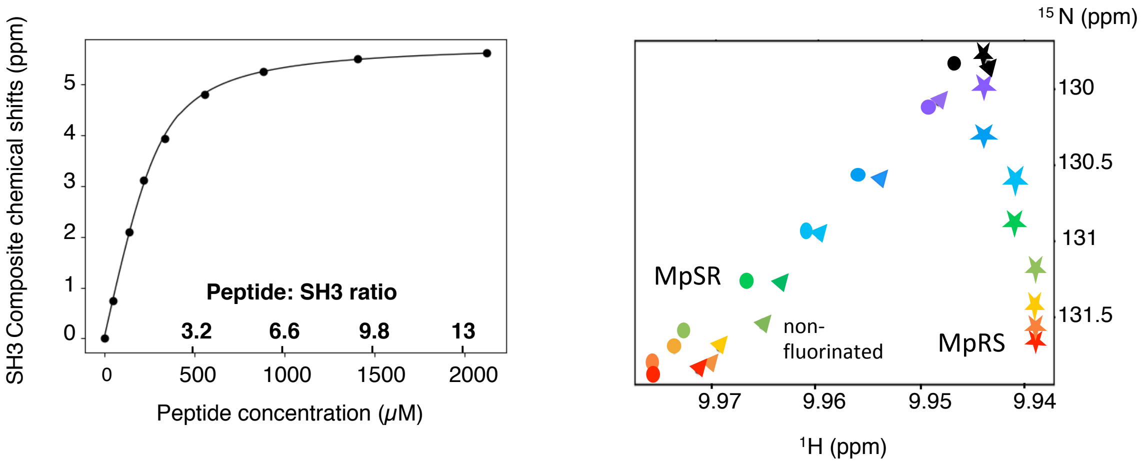

Supplementary Figure 4: Left: ^{1}H, ^{15}N composite chemical shift measured in the ^{1}H-^{15}N HSQC spectra during the titration of the Vinexin β SH3.3 domain with a non-fluorinated model peptide. The fit of the values with a Langmuir model is shown by a solid line and resulted into a dissociation equilibrium constant of 75 ± 5 μM. Right: Position of the Trp 37 Hn-Nε cross peak during the titration. The MpSR and MpRS fluorinated peptides are indicated by discs and stars, respectively while the cross peaks corresponding to the non-fluorinated peptide are show by triangles.

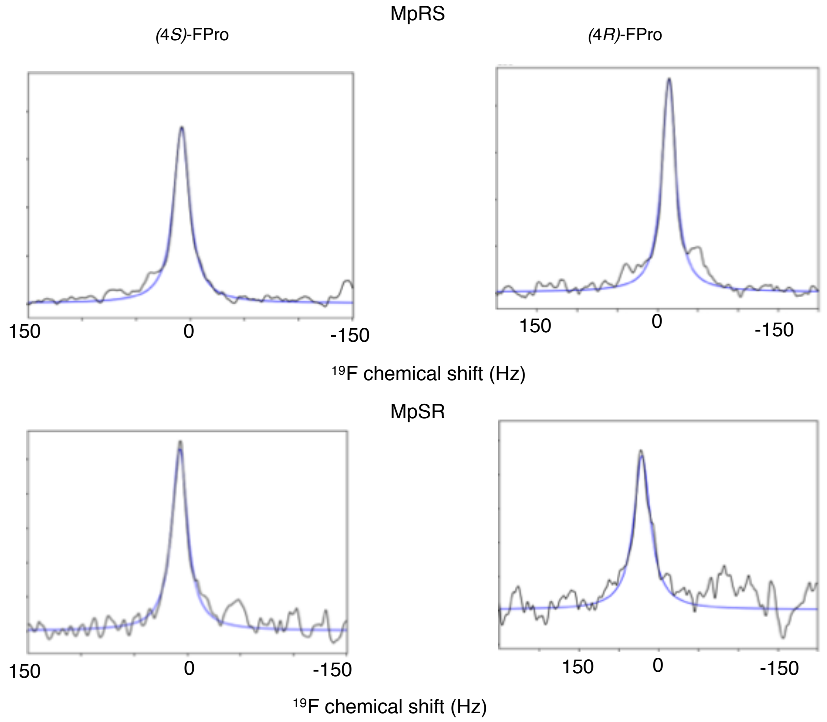

Supplementary Figure 5: Fit of the signals corresponding to (4*S*)-FPro (left) and (4*R*)-FPro (right) residues in the MpRS (top) and MpSR (bottom) peptides at the first titration point. Concentrations are 55.6μM and 50 μM of of MpRS and MpSR respectively. The spectra were recorded at 298K, on a 600 MHz in a 3 mm tube with 2048 scans. Measured signal to noise ratios are 71 for MpRS and 20 for MpSR, after applied apodization of 16 Hz. The fitted linewidhts of (4*S*)-FPro are 12.95 Hz and 12.66 Hz in MpRS and MpSR respectively leading to R_2 values of 40.7 s^{-1} and 39.8 s^{-1}. For (4*R*)-FPro, the linewdiths are 15.6 Hz and 20.24 Hz and the corresponding R2 are 49.3 s^{-1} and 63.6 s^{-1} in MpRS and MpSR respectively.

	K_d (μM)		
	MpRS	MpSR	Non-fluorinated peptide
Gln 14	223 ± 5	76 ± 2	119 ± 30
Asn 15	219 ± 2	80 ± 2	61 ± 2
Asp 17	219 ± 5	73 ± 2	71 ± 2
Leu 21	209 ± 7	73 ± 2	70 ± 4
Trp 37	216 ± 2	73 ± 2	70 ± 3
Val 39	219 ± 4	75 ± 2	69 ± 2
Gly 49	223 ± 8	70 ± 4	53 ± 3
Thr 50	227 ± 4	73 ± 2	72 ± 3
Val 56	218 ± 2	80 ± 5	56 ± 3

Supplementary Table 1: Dissociation equilibrium constants measured for the MpRS, MpSR and non-fluorinated peptide at 298 K in 40 mM phosphate buffer at pH 7. These constants have been obtained by fitting the variations of individual H_N-^{15}N cross-peaks positions upon addition of peptide with a 1:1 equilibrium model. The uncertainties are obtained from the covariance matrix of the fit and do not take in consideration uncertainties on peptide and protein concentrations.

65

	MpRS	MpSR	Non-fluorinated peptide
Gln 14	1.863	1.927	1.949
Asn 15	1.079	1.126	1.093
Asp 17	2.220	2.286	2.276
Leu 21	1.002	0.983	0.988
Trp 37	1.870	2.012	2.011
Val 39	3.094	3.198	3.214
Gly 49	0.800	0.788	0.812
Thr 50	1.841	1.807	1.881
Val 56	1.319	1.310	1.319

Supplementary Table 2: ^{1}H-^{15}N Composite chemical shift perturbation measured for Vinexin β residues that the most affected by the addition of polyproline model peptides.
